# Gambling-like gaming, offline leisure, and help-seeking in problematic gaming among Flemish adolescents: a three-wave longitudinal study

**DOI:** 10.3389/fpsyg.2026.1831367

**Published:** 2026-06-03

**Authors:** Maya Geudens, Lowie Bradt, Rozane De Cock, Bieke Zaman, Eva Grosemans

**Affiliations:** 1 Media, Culture and Policy Lab, Department of Communication Sciences, KU Leuven, Leuven, Belgium; 2 Centre of Expertise Inclusive Society, UCLL Research and Expertise, UC Leuven-Limburg, Heverlee, Belgium; 3 Department of Developmental, Personality and Social Psychology, Ghent University, Ghent, Belgium; 4 Meaningful Interactions Lab, KU Leuven, Leuven, Belgium

**Keywords:** adolescents, gambling-like gaming features, help-seeking, longitudinal study, offline leisure, problematic gaming

## Abstract

**Introduction:**

Problematic gaming among adolescents has received growing attention, yet longitudinal evidence that can inform harm-reduction responses remains limited, particularly regarding gambling-like gaming features, offline leisure, and help-seeking.

**Methods:**

This study examined the prevalence, course, and prospective correlates of problematic gaming in a three-wave annual cohort of Flemish adolescent gamers. Secondary school students who reported gaming at least occasionally completed surveys at baseline (T1: *N =* 1,863), one-year follow-up (T2: *N =* 1,807), and two-year follow-up (T3: *N =* 1,748). Problematic gaming was assessed with the Gaming Disorder Scale for Adolescents. Baseline predictors included engagement with gambling-like gaming features, microtransactions, extracurricular hobby participation, and perceived competence in extracurricular domains; help-seeking knowledge and behavior were assessed at T3.

**Results:**

Problematic gaming was uncommon across waves (1.5–1.9%) and among adolescents who participated at all three waves, none met the cut-off at every assessment. After adjustment for baseline symptoms and sociodemographic covariates, greater engagement with gambling-like gaming features predicted higher problematic gaming symptoms over 1 year, but not over 2 years. Microtransaction engagement was positively correlated with problematic gaming but did not independently predict later symptom change. Neither extracurricular hobby participation nor perceived competence in extracurricular domains were associated with subsequent changes in problematic gaming symptoms. At T3, 53.4% of adolescents reported knowing where they could seek help for gaming-related problems, whereas only 3.5% reported having sought help, and baseline problematic gaming did not prospectively predict later help-seeking.

**Discussion:**

Overall, problematic gaming in this community sample was relatively uncommon and showed limited categorical stability over time. The findings identify gambling-like gaming features as a relevant prospective target for harm-reduction-oriented prevention, whereas the baseline offline leisure indicators did not prospectively predict symptom change. They also point to a gap between perceived access to support and actual help-seeking.

## 1 Introduction

Video gaming is a widespread and often enjoyable leisure activity among adolescents. International estimates suggest that approximately 75–85% of youth play video games (Labrador et al., 2023), and many spend substantial time gaming without experiencing clear problems (Skripkauskaite et al., 2022). Gaming is not inherently harmful and may serve recreational, social, and emotional functions for young people (Barr and Copeland-Stewart, 2022; Chaarani et al., 2022; Raith et al., 2021). At the same time, a smaller subgroup develops problematic gaming patterns marked by functional impairment, with meta-analytic evidence suggesting prevalence estimates of around 3% in the general population and often higher rates in adolescent samples (Satapathy et al., 2025; Stevens et al., 2021). Adolescence is a particularly relevant developmental period in this regard, given heightened sensitivity to rewards and an increased propensity for risk-taking during this life stage (Sisk and Gee, 2022). Problematic gaming has been linked to sleep disruption, academic underperformance, and internalizing symptoms (Kristensen et al., 2021; Richard et al., 2020). However, prevalence estimates vary widely across studies and populations, partly because they depend on how problematic gaming is conceptualized and measured (Kim et al., 2022). International prevalence figures should therefore be compared with caution. This also highlights the need for up-to-date estimates in Flemish adolescents.

Prevalence estimates, however, do not explain why some adolescents develop problems whereas others do not. Addressing questions of onset and escalation therefore requires attention to risk and protective factors, especially because problematic gaming cannot be reduced to gaming intensity alone (Warburton et al., 2022). Socio-ecological perspectives suggest that gaming-related harm emerges from interacting influences at multiple levels rather than from individual vulnerability alone (Geudens et al., 2026; Mihara and Higuchi, 2017; Zhuang et al., 2023), and prior research has linked problematic gaming to a range of individual and environmental influences (Jeong et al., 2021; Paulus et al., 2018; Zhuang et al., 2023). Yet, much of this evidence remains cross-sectional, which limits conclusions about temporal ordering (Zhuang et al., 2023). In addition, quantitative research has focused predominantly on intra-individual characteristics, with comparatively less attention to interpersonal, environmental, and medium-related determinants (Mihara and Higuchi, 2017).

From a harm-reduction perspective, it is useful to distinguish between influences related to the gaming environment itself, the individual, and the broader social context, because this helps identify modifiable sources of risk and protection and points to different targets for prevention, regulation, and support (Popelier et al., 2017). Zinberg’s agent–set–setting framework offers a useful lens for doing so, as it conceptualizes harmful versus controlled engagement as the outcome of an interaction between the activity or product itself (“agent”), the individual (“set”), and the social and physical context (“setting”) (Dalgarno and Shewan, 2005; Zinberg, 1984). In gaming, agent-related influences include medium-specific design affordances, such as monetization structures and chance-based reward mechanics, which may shape engagement and constitute potential targets for prevention, regulation, and safer design. In the present study, we use this framework to organize our candidate predictors: gambling-like gaming features and microtransactions are treated as agent-related influences, perceived competence in extracurricular domains as a set-related factor, and offline hobby participation and help-seeking as setting-related factors. In doing so, it aims to clarify which influences may be most relevant for harm-reduction-oriented prevention and early support in adolescence.

### 1.1 Understanding problematic gaming: risk and protective factors

One particularly relevant agent-level domain concerns gambling-like and monetization-related features embedded in contemporary games. Cross-sectional studies consistently report associations between adolescents’ engagement with gambling-like features and elevated levels of problematic gaming (Chang et al., 2024; Hing et al., 2023). However, the available evidence remains limited and is predominantly cross-sectional, restricting conclusions about temporal ordering (Rehbein et al., 2010; Saini and Hodgins, 2023). This has led to explicit calls for more research using interactionist frameworks that examine how player characteristics and game design features jointly contribute to dysregulated gaming patterns (Flayelle et al., 2025). Although some longitudinal studies have begun to examine specific mechanics such as loot-box purchasing, follow-up periods are often short and broader multi-wave tests remain scarce (González-Cabrera et al., 2023). In addition, the literature has predominantly focused on isolated features—especially loot boxes—rather than on the broader combinations of gambling-like and monetization-related elements that adolescents are likely to encounter in contemporary games (Gibson et al., 2022; Montiel et al., 2022; Siste et al., 2025). Yet these features often co-occur within wider monetized gaming environments, suggesting that a narrow focus on single mechanics may provide only a partial view of adolescents’ exposure (Denoo et al., 2025; King and Delfabbro, 2020).

In the present study, we adopt a broader exposure perspective. However, there is no consensus on terminology for gambling-related features in digital games. Labels such as simulated gambling, virtual gambling, gamble-play media, and non-monetary gambling are used inconsistently across the literature (Dupont et al., 2024). Following Declerck (2025), we use the umbrella term *gambling-like gaming* to refer to gambling-adjacent features in digital games, without implying that these features should necessarily be classified as formal gambling in a field marked by ongoing legal and conceptual debate. Such features have been argued to share key structural characteristics with monetary gambling, including chance-based rewards and reinforcement dynamics (Griffiths and Nuyens, 2017; King et al., 2015). In this study, gambling-like gaming is operationalized as adolescents’ engagement over the past 12 months with common gambling-adjacent features in games, including acquiring and purchasing loot boxes, selling loot box contents, playing social-casino style games, and using chance-based spin mechanics. We also consider engagement with microtransactions, defined as purchases made with real money, or with premium in-game currency bought with real money, for virtual goods or services such as cosmetic items, additional content, season passes, or gameplay advantages. Because these purchasing features often co-occur with gambling-like elements within broader monetized gaming environments (King and Delfabbro, 2020), examining them together provides a broader view of adolescents’ exposure to interconnected monetization-related features, rather than focusing on a single mechanic in isolation (Denoo et al., 2025).

Potentially relevant influences are not limited to game design. Adolescents’ offline leisure lives may shape how central gaming becomes in everyday life. One possible rationale is that time and investment devoted to gaming may reduce engagement in other developmentally meaningful activities, an idea often discussed in terms of displacement (Lee, 2009). Gaming unfolds within socio-ecological contexts (e.g., family, school, peers, clubs) that can either heighten vulnerability or provide alternative sources of structure and belonging (Bronfenbrenner, 1977; Mihara and Higuchi, 2017; Zhuang et al., 2023). Some emerging longitudinal evidence suggests that higher levels of physical activity may be associated with a lower risk of later gaming-related problems (Nowotarski et al., 2024). At the same time, offline leisure is frequently promoted in prevention as a protective strategy by emphasizing meaningful alternatives to gaming (American Academy of Pediatrics, 2026; Stevens et al., 2021). However, empirical support for offline leisure as a protective factor remains limited and much of the available research is still cross-sectional. Existing studies also tend to focus on specific activities, rather than examining broader patterns of offline engagement across leisure domains (Nowotarski et al., 2024; Ropovik et al., 2023; Zhuang et al., 2023).

In addition, participation in offline leisure activities alone may not capture whether offline contexts function as meaningful alternatives to gaming. Offline activities may be particularly relevant when they provide adolescents with experiences of competence, which could reduce reliance on gaming as a primary source of achievement and recognition (Ryan and Deci, 2000). Yet this dimension has received even less attention in empirical research than activity participation itself (Zhuang et al., 2023). The present study therefore distinguishes between two aspects of adolescents’ offline lives at baseline: the number of offline hobby activities in which they participate, and their perceived competence in extracurricular domains such as sports, music, and creativity.

A further question concerns whether adolescents with higher levels of problematic gaming are connected to potential sources of support. From a harm-reduction perspective, help-seeking matters because timely support may prevent gaming-related problems from becoming more impairing. Yet in youth mental health and addictions, recognizing a need for help, knowing where to go, and actually using services often do not coincide (Degenhardt et al., 2017; Wang et al., 2004). In adolescent behavioral and mental health care, pathways into support are often adult-mediated, and self-recognition does not reliably translate into service use (Bore et al., 2025; Zwaanswijk et al., 2003). Emerging work in gaming disorder likewise suggests that adolescents at elevated risk may not be more inclined to seek professional help than their peers (Jeon et al., 2022; Yu et al., 2024). However, the evidence remains limited and is drawn largely from East Asian contexts and treatment-seeking samples, restricting generalizability to community adolescents in Western European settings.

In response to these limitations in previous research, the present study uses data from three annual waves in a large Flemish school cohort. Specifically, the study addresses four questions. First, we provide method-comparable estimates of problematic gaming prevalence, mean-level change, and temporal stability in an under-documented Western European context (RQ1). Second, we examine whether baseline engagement with gambling-like gaming and microtransactions predicts subsequent change in problematic gaming symptoms over one- and two-year intervals (RQ2). Third, we investigate whether two indicators of adolescents’ offline lives—breadth of participation in offline hobby activities and perceived competence in extracurricular domains—are associated with baseline levels and subsequent change in problematic gaming symptoms (RQ3). Fourth, we examine whether adolescents know where they could seek help and whether they have sought help for gaming-related problems, thereby distinguishing perceived help pathways from actual help-seeking behavior. We also examine whether baseline symptom levels are prospectively associated with subsequent help-seeking (RQ4).

## 2 Materials and methods

### 2.1 Procedure

Ethical approval for the study was obtained from the KU Leuven Social and Societal Ethics Committee [SMEC; reference no. G-2021-3439-R2(AMD)m]. Data were collected at three measurement points across three consecutive academic years in a sample of secondary schools in Flanders, the northern Dutch-speaking region in Belgium. The first data gathering, hereafter referred to as T1, took place between November 2021 and February 2022, with two additional data collections administered at roughly one-year intervals. The second and third measurement points, T2 and T3 respectively, also took place between November and February in the subsequent years. The study forms part of a larger interdisciplinary project on gaming, gambling and mental health in adolescence.

Before the baseline (T1) assessment, schools were approached through an informational letter and subsequently recontacted by email or telephone to confirm participation. School selection focused on ensuring variation in educational tracks within the Flemish secondary education system (which comprises a general/academic track, a technical track, a vocational track, and an arts track, typically enrolling pupils aged 12–18) and the inclusion of pupils from the first year of secondary education onward, so that participants could be followed longitudinally across multiple waves. School recruitment aimed to capture heterogeneity across school contexts and educational tracks, rather than to achieve proportional representativeness at the school or regional level. School principals provided written consent on behalf of the institution; following school approval, parents or legal guardians were informed and could decline their child’s participation via passive consent procedures. Pupils were briefed on the study’s aims and data collection procedures and were reminded that participation was voluntary and that they could discontinue at any point. Recruitment was not restricted based on gaming behavior; all enrolled pupils were eligible. However, only pupils who indicated that they played video games at least occasionally were presented with the gaming-related section of the questionnaire and are included in the present analyses.

Most respondents completed the questionnaire online during regular class hours using school computers. In a subset of schools, the survey link was distributed through the school’s digital learning platform and completed at home. All materials were administered in Dutch. The survey required approximately 30 min to complete at each measurement point.

To enable accurate linkage of participants’ responses across measurement waves, pupils provided identifying information during data collection, including their name and class. These identifiers were used solely for longitudinal matching. After data linkage, personal identifiers were replaced by random numerical codes. The linkage key connecting identifiers to codes was stored securely and separately from the analytical dataset, which contained coded participant records only.

A subset of the measures used in this study has been employed in previous publications stemming from the same broader research project Game(e)(a)ble. Specifically, earlier cross-sectional and longitudinal analyses have used the items on gambling-like elements in video games and the GADIS-A indicators of problematic gaming. The present study, however, addresses distinct research questions -- specifically, the longitudinal prediction of changes in problematic gaming from medium-related (gambling-like gaming features, microtransactions) and environment-related (extracurricular engagement and perceived competence) variables, as well as help-seeking patterns -- that have not been analyzed or reported elsewhere.

### 2.2 Participants

Participants provided data on background variables via self-report measures at each measurement point. Sociodemographic characteristics for the full sample at T1, T2, and T3 are presented in Table 1. For the prospective analyses, we used the subset of adolescents who participated at baseline and completed the problematic gaming measure at least once at follow-up; characteristics of this longitudinal subsample are also shown in Table 1.

**Table 1:** Sociodemographic characteristics of participants by measurement wave and longitudinal subsample.

Characteristic	T1	T2	T3	Longitudinal subsample
*N*	1,863	1,807	1,748	889
Age, M (SD)	14.00 (1.45)	14.31 (1.83)	15.18 (1.88)	13.79 (1.33)
Boys, %	53.0	54.7	57.7	51.9
Two-parent family, %	72.5	71.4	71.5	75.7
Family manages easily/very easily, %	72.4	64.6	71.0	78.0
Academic track, %	72.9	69.4	64.0	82.5
Technical track, %	16.0	17.1	21.1	14.1
Vocational track, %	8.1	11.1	11.0	3.0
Arts track, %	2.0	1.3	3.9	0.2

Values are percentages unless otherwise indicated. Age is presented as mean (standard deviation). Percentages may not sum to 100 because of rounding and missing or other responses. The longitudinal subsample includes adolescents who participated at baseline and completed the problematic gaming measure at least once at follow-up.

### 2.3 Measures

*Problematic gaming*: problematic gaming was assessed using the Dutch, validated adaptation (Grosemans et al., 2025) of the 9-item Gaming Disorder Scale for Adolescents (GADIS-A; Paschke et al., 2020). Participants rated how strongly each statement applied to them during the last 12 months on a five-point Likert scale ranging from 1 (completely disagree) to 5 (completely agree). Items capture cognitive-behavioral symptoms and negative consequences of gaming (e.g., “I continue to play games even though it negatively affects my performance at school, during my internship, or at work.” and “I neglect my daily tasks because I prefer to play games.”). Internal consistency of all the items was very good at all three measurement points, with Cronbach’s alphas of 0.85 at Wave 1, 0.86 at Wave 2, and 0.87 at Wave 3.

We operationalized problematic gaming in two ways. First, we used a continuous score for each participant at each measurement point, computed as the mean of all nine GADIS-A items. Second, we derived a clinical cut-off classification distinguishing adolescents with problematic versus non-problematic gaming at each measurement point. Following the scoring guidelines proposed for the GADIS-A (Grosemans et al., 2025; Paschke et al., 2020), adolescents were classified as meeting the threshold for problematic gaming when they simultaneously exceeded (a) a mean score of > 2.00 on the four negative consequences items, (b) a mean score of > 3.34 on the five cognitive–behavioral symptoms items, and (c) a mean score of > 2.23 across all nine items. In addition, participants answered an item assessing how often they had experienced problems due to gaming in the past year on a four-point Likert scale, in which they were required to report “during extended periods” or “almost daily” on this item to meet the problematic gaming cut-off.

*Environment: engagement in extracurricular activities*: at the first timepoint, adolescents reported their participation in extracurricular activities by indicating whether they engaged in several hobby categories (e.g., being a member of a sports club, exercising regularly outside of a club, participating in a youth organization, playing a musical instrument or attending music classes, engaging in creative hobbies such as drawing or coding). Response options allowed participants to select all activities that applied, as well as indicating “none of the above”. Based on these responses, we constructed a three-level index reflecting the breadth of extracurricular engagement: 0 (no extracurricular activities), 1 (engagement in one activity), and 2 (engagement in two or more activities).

*Environment: perceived competence in extracurricular domains:* perceived competence in extracurricular domains was measured at the first timepoint using three items assessing how adolescents evaluated their own abilities relative to their peers in sports, music, and creative activities. For each domain, participants provided a rating on a continuous slider ranging from 0 (“much worse than peers”) to 100 (“much better than peers”). This composite was intended as a pragmatic index of broader perceived offline competence, rather than as evidence that sport, music, and creative competence form a single unidimensional construct. To create a standardized indicator of perceived competence, scores on all three domains were first converted into z-scores. A composite perceived competence score was then computed as the mean of the three standardized domain scores, with higher values indicating higher self-evaluated competence across extracurricular domains.

*Medium: engagement with gambling-like elements in video games:* at the first measurement point, adolescents indicated how often they had engaged in a set of five gambling-like features embedded in video games during the past 12 months, using a 7-point frequency scale (1 = never, 2 = a few times a year, 3 = once a month, 4 = a few times a month, 5 = once a week, 6 = a few times a week, 7 = every day). These behaviors included acquiring free loot boxes, purchasing loot boxes with real money, selling loot box contents, skin betting, playing social casino games and spinning in-game wheels for in-game rewards. Responses were aggregated into a scale score reflecting overall engagement with gambling-like game elements in video games. Internal consistency of this scale was good, with a Cronbach’s alpha of 0.75.

*Medium: engagement with microtransactions:* engagement with in-game microtransactions was measured at the first wave using two self-report indicators. Participants reported how frequently they purchased gaming-related items (e.g., cosmetic skins) via microtransactions on a five-point scale ranging from never to often. They also indicated their approximate monthly spending on microtransactions in euros. To align spending with the frequency scale, monthly expenditure was recoded into five ordered of categories (0; 1–9; 10–29; 30–99; ≥100 euros). A composite index was computed by averaging the frequency and spending indicators. Because the measure consisted of two items, internal consistency was evaluated using the inter-item Pearson correlation (r = 0.31) and was adequate.

*Help-seeking:* help-seeking related to gaming problems was assessed at the third measurement point using two dichotomous items. Participants indicated (0 = no, 1 = yes) (a) whether they knew where to go if they needed help for gaming-related difficulties and (b) whether they already sought help from someone in their environment for problems related to gaming.

### 2.4 Plan of analysis

Analyses were conducted in several steps, corresponding to the study’s research questions. All analyses were performed in R. First, to address RQ1, we computed descriptive statistics for problematic gaming (both with a cut-off score and a continuous score) and help-seeking at each measurement point. For the continuous problematic gaming scores, we examined whether individual adolescents showed meaningful changes in their problematic gaming levels over time. To do so, we estimated a linear mixed-effects model with random intercepts for participants. This model captures whether individual adolescents’ scores increase or decrease over time, accounting for between-person differences in baseline levels. In addition to examining within-person change, we also compared the average level of problematic gaming across the full sample at each measurement point. Follow-up pairwise comparisons between waves were conducted using estimated marginal means with Holm-adjusted *p*-values. These comparisons test whether the mean level of problematic gaming in the group at the first measurement point differs from the mean at the second and the third measurement point, regardless of whether individual adolescents personally increased or decreased.

Second, to address RQ2 and RQ3, we restricted the sample to adolescents who provided baseline (T1) data on all medium-related (i.e., Engagement with Gambling-like Elements in Video Games and with Microtransactions) and environment-related (i.e., engagement and perceived competence in extracurricular activities predictors) and who also completed the problematic gaming questionnaire at least once at a later measurement point. Prior to running the longitudinal models, we computed descriptive statistics and bivariate associations between all background, medium and environment variables and problematic gaming across all three measurement points.

Next, multivariate analyses of variance (MANOVAs) were conducted with all background variables (age, gender, educational track, family structure, and perceived financial comfort) entered simultaneously as predictors and all study variables as outcomes. Background variables showing significant multivariate effects were retained as covariates for the longitudinal analyses.

To examine longitudinal effects of medium and environment variables on changes in problematic gaming, two autoregressive regression models were estimated (i.e., models in which the outcome at a later time point was predicted by scores on the same variable at the preceding time point, thereby predicting change over time). The first model predicted problematic gaming at the second measurement point from all predictors while controlling for baseline levels; the second did so for the third measurement point. By including baseline problematic gaming as a predictor, these models estimate unique associations between the medium and environment variables and changes in problematic gaming over one- and two-year intervals, adjusted for the effects of the other predictors. In a final step, interaction terms between gender and each medium variable were added to both models to test whether effects on changes in problematic gaming differed for boys and girls.

## 3 Results

### 3.1 Prevalence of problematic gaming and help-seeking

To examine how common problematic gaming was in this cohort, whether mean symptom levels changed over time, and how stable problematic gaming was across three annual waves, all adolescents who filled in the relevant surveys are included in the analyses. At the first measurement point, 33 out of 1863 adolescents who played video games (1.8%) scored above the problematic gaming cut-off. At the second measurement point, 35 out of 1807 participants (1.9%) exceeded the cut-off, and at the third measurement point, 27 out of 1748 participants (1.5%) met the threshold. Out of the 344 adolescents who participated in the problematic gaming survey at all three measurement points, 14 scored above the problematic gaming cut-off at least at one measurement point (4.1%), and 3 of them at two measurement points (0.9%). None of them met the threshold at all measurement points.

In terms of the continuous score on problematic gaming, a linear-mixed effects model showed that problematic gaming changed significantly within participants over time (*F*(2, 2919.5) = 17.68, *p <* 0.001), indicating that individuals’ scores were not stable across measurement points. Pairwise comparisons of the group means showed that the average level of problematic gaming at T1 (M = 1.88) was significantly higher than at T2 (M = 1.80; *p <* 0.001) and T3 (M = 1.80; *p <* 0.001). However, the mean scores at T2 and T3 did not differ significantly from each other (*p* = 0.30).

To examine whether adolescents with higher levels of problematic gaming symptoms knew where to seek help and reported having sought help for gaming-related problems, help-seeking was examined at the third measurement point. At T3, 933 adolescents (53.4%) reported knowing where they could seek help if they experienced gaming-related problems, whereas 61 adolescents (3.5%) reported that they had already sought help. Out of these 61 who indicated to have sought help, 6 (9.8%) scored above the problematic gaming cut-off at the third measurement point. None of them scored above the problematic gaming cut-off at T1 or on T2. Overall, these descriptive findings suggest that knowledge of where to seek help was relatively common, whereas actual help-seeking was rare.

### 3.2 Longitudinal analyses: descriptive statistics and correlations

As a first step in examining whether medium-related and environment-related factors were linked to problematic gaming over time, analyses were restricted to adolescents who completed baseline (T1) data on these predictors and who also completed the problematic gaming measure at T1 and at least one follow-up assessment at T2 or T3. Associations between background, medium-, and environment-related variables and problematic gaming scores across measurement points were then examined using Spearman rank-order correlations because several variables deviated from normality.

Correlations between background, environment, medium variables, and continuous scores on problematic gaming are presented in Table 2. Problematic gaming scores were moderately stable across waves, with significant positive associations between T1 and T2 (*ρ* = 0.52, *p <* 0.001), T1 and T3 (*ρ* = 0.50, *p <* 0.001), and T2 and T3 (*ρ* = 0.60, *p <* 0.001).

**Table 2:** Descriptive statistics and spearman correlations between study and background variables.

Variable	M	SD	1	2	3	4	5	6	7	8	9	10	11	12	13
Problematic gaming – continuous scores															
1. T1 - Problematic Gaming	1.83	0.63													
2. T2 – Problematic Gaming	1.72	0.63	0.52***												
3. T3 – Problematic Gaming	1.71	0.64	0.50***	0.60***											
Background variables															
4. Age	13.79	1.33	−0.09**	−0.04	−0.10*										
5. Gender – Boys	0.52	0.50	0.26***	0.26***	0.37***	0.02									
6. Education – Academic Track	0.82	0.38	−0.12***	−0.05*	−0.03	−0.14***	−0.05								
7. Family Status – Two-parent Family	0.76	0.43	−0.07*	−0.08*	0.02	0.01	0.05	0.09*							
8. Socio-Economic Status – Perceived Financial Comfort	4.98	0.84	−0.14***	−0.10*	−0.07	−0.02	0.01	0.13***	0.20***						
Environment variables															
9. T1 - Engagement in Hobbies	1.53	0.62	−0.10**	0.00	−0.03	0.04	−0.02	0.11***	0.12***	0.17***					
10. T1 - Perceived Competence in Hobbies	−0.02	0.56	−0.02	−0.05	−0.03	−0.04	0.00	0.06	0.07*	0.15***	0.18***				
Medium variables															
11. T1 - Engagement with Gambling-like Gaming Features	1.74	0.82	0.34***	0.32***	0.32***	−0.02	0.37***	−0.03	−0.05	−0.03	0.02	0.01			
12. T 1- Engagement with Microtransactions	1.55	0.83	0.37***	0.26***	0.30***	−0.12***	0.42***	−0.08*	−0.08*	−0.05	−0.10**	−0.02	0.40***		
Help-seeking variables															
13. T3 - Help-seeking: Knowledge	0.50	0.50	0.03	0.01	0.02	−0.03	0.07	−0.03	0.08*	−0.02	−0.06	−0.03	0.11**	0.07	
14. T3 - Help-seeking: behavior	0.01	0.10	0.05	−0.01	0.09*	−0.06	0.06	−0.04	−0.06	−0.01	0.08	0.00	0.07	0.08*	0.03

^*^p < 0.05; ^**^p < 0.01; ^***^p < 0.001.

With respect to background variables, higher problematic gaming scores were consistently associated with being male at all three measurement points (*ρ*s = 0.26–0.37, ps < 0.001). Older age showed small negative associations with problematic gaming at T1 and T3 (*ρ* = −0.09, *p <* 0.01 and −0.10, *p <* 0.05, respectively). Being enrolled in the academic track (*ρ* = −0.12, *p <* 0.001), living in a two-parent family (*ρ* = −0.07, *p <* 0.05), and higher perceived financial comfort (*ρ* = −0.14, *p <* 0.001) were each negatively correlated with problematic gaming at baseline, with less consistent associations at later waves.

Regarding the environment-related variables, baseline engagement in extracurricular activities was negatively correlated with problematic gaming at T1 (*ρ* = −0.10, *p <* 0.01), but not at T2 or T3. It was also positively associated with enrollment in the academic track (*ρ* = 0.11, *p <* 0.001), living in a two-parent family (*ρ* = 0.12, *p <* 0.001), and higher perceived financial comfort (*ρ* = 0.17, *p <* 0.001). Perceived competence in extracurricular domains was not significantly associated with problematic gaming at any time point.

Concerning medium-related variables, engagement with gambling-like elements in video games at T1 was moderately positively associated with problematic gaming at T1 (*ρ* = 0.33, *p <* 0.001), T2 (*ρ* = 0.32, *p <* 0.001), and T3 (*ρ* = 0.31, *p <* 0.001). These were among the strongest bivariate associations in the correlation matrix. Similarly, engagement with microtransactions at T1 was positively correlated with problematic gaming across all three waves (*ρ*s = 0.26–0.37, ps < 0.001).

Finally, help-seeking knowledge at T3 showed a small positive association with gambling-like gaming (*ρ* = 0.11, *p <* 0.01). Help-seeking at T3 showed only small and limited correlations, including with problematic gaming at T3 (*ρ* = 0.09, *p <* 0.05) and with microtransaction engagement reported in the baseline assessment (*ρ* = 0.08, *p <* 0.05).

### 3.3 Longitudinal analyses: autoregressive regression models predicting changes in problematic gaming

We next tested whether medium- and environment-related factors predicted subsequent changes in problematic gaming over one- and two-year intervals. To decide which background variables to control for in the final analyses, multivariate analyses with all background variables (adolescents’ gender identity, age, educational level, family structure and the perceived financial comfort of their family) as predictors and all study variables (baseline problematic gaming, engagement with gambling-like gaming elements, engagement with microtransactions, engagement in extracurricular activities, and perceived competence in extracurricular domains) as outcomes were performed. Significant multivariate effects were found for all background variables, age [Wilks’ *Λ* = 0.98, *F*(5, 828) = 4.24, *p <* 0.001], gender [Wilks’ Λ = 0.83, *F*(5, 828) = 33.81, *p <* 0.001], type of education [Wilks’ Λ = 0.97, *F*(5, 828) = 5.98, *p <* 0.001], family structure [Wilks’ Λ = 0.97, *F*(5, 828) = 4.93, *p <* 0.001], and household income [Wilks’ Λ = 0.94, *F*(5, 828) = 11.21, *p <* 0.001].

Next, longitudinal analyses were performed to test the effects of medium and environment variables on changes in problematic gaming using autoregressive regression models. Two sets of hierarchical linear regression models were tested; one predicting changes in problematic gaming from the first to the second timepoint, and one predicting changes in problematic gaming from the first to the third timepoint. By including baseline problematic gaming as a covariate, these models estimated the unique associations between baseline predictors and subsequent changes in problematic gaming. Given mild deviations from normality, robust standard errors (HC3) were used to calculate *p*-values. In each set of models, background variables and baseline problematic gaming were entered first, followed by the environment variables (Step 2a) and the medium variables (Step 2b). In Step 3, both sets of predictors were included simultaneously, and in Step 4 the two gender-by-medium interaction terms were added.

For the prediction of changes from the first to the second measurement point, engagement with gambling-like elements in video games showed a small but significant positive effect across steps, whereas none of the other environment or medium variables were associated with changes in problematic gaming. Boys also showed greater increases in problematic gaming. For the prediction of changes from the first to the third measurement point, none of the environment or medium variables were significant predictors, while boys again exhibited larger increases. No significant interaction effects between gender and the medium variables emerged. Full model estimates are presented in Table 3.

**Table 3:** Coefficients of longitudinal hierarchical regression analyses predicting changes in problematic gaming over 1- and 2-year intervals.

Predictor / model term	Change in problematic gaming from T1 to T2					Change in problematic gaming from T1 to T3				
	Step 1	Step 2a	Step 2b	Step 3	Step 4	Step 1	Step 2a	Step 2b	Step 3	Step 4
Background variables and baseline problematic gaming										
T1 - Problematic Gaming	0.51***	0.51***	0.48***	0.48***	0.47***	0.42***	0.42***	0.40***	0.40***	0.40***
Age	0.02	0.02	0.02	0.02	0.01	−0.05	−0.05	−0.05	−0.05	−0.05
Gender – Boys	0.13***	0.13***	0.10*	0.10*	0.09*	0.24***	0.24***	0.22***	0.22	0.21***
Education – Academic Track	0.02	0.01	0.02	0.02	0.01	0.02	0.02	0.02	0.02	0.02
Family Status – Two-parent Family	−0.03	−0.04	−0.02	−0.03	−0.03	0.04	0.04	0.04	0.04	0.04
Socio-Economic Status – Perceived Financial Comfort	−0.06	−0.06	−0.06	−0.06	−0.06	−0.02	−0.02	−0.03	−0.03	−0.02
Environment variables										
T1 - Engagement in Hobbies		0.05		0.05	0.05		0.00		0.00	0.00
T1 - Perceived Competence in Hobbies		−0.02		−0.02	−0.02		0.00		0.00	−0.01
Medium variables										
T1 - Engagement with Gambling-like Gaming Features			0.10*	0.10*	−0.02			0.04	0.04	0.06
T1 - Engagement with Microtransactions			0.02	0.02	0.08			0.02	0.02	0.12
Gender x medium interactions										
Gender x Engagement with Gambling-like Elements in Video Games					0.01					−0.02
Gender x Engagement with Microtransactions					−0.04					−0.11
*R*^2^ Change	0.31***	0.00	0.01*	0.01* / 0.00	0.00	0.28***	0.00	0.00	0.00/0.00	0.00

^*^p < 0.05; ^**^p < 0.01; ^***^p < 0.001. Standardized regression coefficients are reported. *P*-values are based on robust standard errors (HC3). Step 2 consists of two substeps. In Step 2a, the environment variables are added to the baseline model, and the corresponding increase in explained variance (ΔR^2^) is reported. In Step 2b, the medium variables are entered instead of the environment variables, with a separate ΔR^2^ indicating their incremental contribution compared to Step 1. Step 3 includes both predictor sets simultaneously. Because this model can be compared against two different Step 2 models, the R^2^ change is presented in two parts. The value before the slash reflects the increase in explained variance when adding the medium variables on top of the environment variables (Step 3 versus Step 2a); the value after the slash reflects the increase when adding the environment variables on top of the medium variables (Step 3 versus Step).

## 4 Discussion

Using three annual waves in a population-based cohort of Flemish adolescents (T1: *N =* 1863; T2: *N =* 1807; T3: *N =* 1748), this study examined the prevalence and course of problematic gaming and tested whether gambling-like gaming, offline leisure, and help-seeking were prospectively associated with symptom change. Overall, our findings show that problematic gaming was relatively uncommon and that gambling-like gaming predicted increases in symptoms at the one-year follow-up but not at the two-year follow-up. Furthermore, the results indicate that baseline offline leisure indicators did not predict later decreases in problematic gaming symptoms in the present models, and that help-seeking appeared limited despite many adolescents reporting that they knew where to turn for support.

### 4.1 Prevalence and temporal stability of problematic gaming

With respect to RQ1, problematic gaming had a low prevalence and limited categorical stability across adolescence. Between 1.5 and 1.9% of adolescent gamers met the GADIS-A cut-off at each wave, and among those who participated at all three waves, 4.1% met the cut-off at least once, whereas none did so consistently across all assessments. In this community sample, problematic gaming therefore appeared less to reflect a stable categorical status than fluctuating symptom severity over time. This suggests that a continuous symptom approach may be more informative than a strict case classification in longitudinal research on adolescent problematic gaming. Accordingly, the findings are best interpreted as indicating change in symptom levels over time rather than stable transitions in case status. However, this interpretation should be made cautiously, as adolescents with higher symptom levels may have been more likely to drop out over time, which could have led to an underestimation of persistence. Even so, prevalence in this Flemish sample was lower than that reported in other adolescent samples using the same scoring algorithm (e.g., approximately 3.7–4.0%) (Nazari et al., 2023; Paschke et al., 2020).

### 4.2 Engagement with gambling-like gaming features as a prospective risk factor for symptom growth

A central finding of this study related to RQ2 is that adolescents who reported greater engagement with gambling-like gaming features at baseline showed higher problematic gaming symptoms 1 year later, even after adjustment for baseline symptoms and relevant background covariates, including age, gender, educational track, family structure, and perceived financial comfort. This prospective association was not observed at the two-year follow-up, suggesting that gambling-like engagement may be more relevant to temporary symptom escalation than to sustained symptom change in this cohort.

This prospective association at the one-year follow-up was not explained by more general spending involvement. Although microtransaction engagement was correlated with problematic gaming at all three waves, it did not independently predict later symptom change once baseline symptoms and gambling-like engagement were taken into account. This suggests that microtransactions may mainly signal broader involvement in monetized games and partly overlap with gambling-like features. Together, these results suggest that the link between baseline engagement with gambling-like gaming features and later increases in problematic gaming symptoms is unlikely to be explained by involvement in monetized gaming more generally. Instead, gambling-like engagement appears to reflect a distinct risk factor related to the gaming medium itself for symptom increases at the one-year follow-up.

We found no evidence that this association differed between boys and girls. This contrasts with González-Cabrera et al. (2023), who found that the prospective association between loot-box purchasing and later gaming-related problems was present for girls, but not for boys.

Our findings extend a literature that has been dominated by cross-sectional studies (Siste et al., 2025) and by a narrow focus on single mechanics such as loot boxes (Gibson et al., 2022; Montiel et al., 2022). Rather than isolating one feature, we examined a broader set of gambling-like affordances alongside microtransactions across three annual waves. The results suggest that gambling-like design features may matter in their own right, rather than simply reflecting generally high involvement in gaming (Delfabbro and King, 2020; Hing et al., 2023; King and Delfabbro, 2020).

Several mechanisms discussed in prior literature may help explain why gambling-like engagement could precede symptom escalation. Chance-based rewards may reinforce repeated play or spending through intermittent reinforcement, and experimental work on loot boxes suggests that they can increase arousal and the urge to continue playing (Brady and Prentice, 2021; Larche et al., 2021). “Near-miss” feedback may likewise prolong play and increase the urge to continue, as near-misses are known to encourage continued engagement in gambling contexts (Dixon et al., 2011). Reward cues may also draw adolescents back into the game and trigger renewed urges to play (Zhang et al., 2016). Games that encourage ongoing spending and progression may also make disengagement more difficult once adolescents have already invested time and money (King and Delfabbro, 2018).

Building on these findings, future longitudinal work could track gambling-like engagement over time, including when adolescents start, continue, reduce, or stop using these features. This would make it possible to compare adolescents with different cumulative exposure patterns—for example, those who are never exposed, briefly exposed, repeatedly exposed, or increasingly exposed—and to examine whether these patterns matter differently in earlier versus later adolescence.

Taken together, these findings suggest that gambling-like engagement should be understood within broader monetized gaming environments rather than only in terms of isolated mechanics.

### 4.3 From single mechanic to clustered exposure

The findings for RQ2 also point to a broader conceptual issue: adolescents are likely to encounter gambling-like features as part of wider monetized gaming environments rather than as single, isolated mechanics. In this study, gambling-like activities tended to co-occur rather than appear as separate behaviors, suggesting that adolescents may be exposed to broader clusters of monetized and gambling-like features rather than to one isolated mechanic. Importantly, this does not imply that specific mechanics are unimportant or harmless. Rather, it suggests that risk may accumulate through repeated exposure to combinations of gambling-like affordances within broader monetized gaming environments.

From this perspective, our findings suggest that the more informative question is not which single mechanic is most harmful in isolation, but how the breadth, intensity, and combination of gambling-like affordances accumulate across adolescents’ gaming trajectories.

From a harm-reduction perspective, this implies that responses should not focus only on individual mechanics. Because gambling-like features often co-occur within broader monetized gaming environments, interventions that target only one mechanic may address only part of the broader risk context. This concern is also reflected in the policy literature, which has argued that regulatory responses should look beyond loot boxes alone and consider broader gambling-like and monetized features in digital games (Cerulli-Harms et al., 2020; Children’s Commissioner for England, 2019). Implementation evidence also indicates that probability disclosure policies may offer only limited protection. For example, research on loot box disclosures has shown inconsistent compliance and poor visibility, suggesting that disclosure alone is unlikely to be sufficient as a safeguard (Xiao and Newall, 2022). Belgium illustrates a different limitation: even in a context where paid loot boxes have been classified as illegal, adolescents still report access and workarounds, suggesting that narrowly targeted bans may be circumvented in practice (Denoo et al., 2023). Taken together, these considerations support prevention and policy approaches that address broader patterns of gambling-like and monetized exposure rather than isolated features.

Building on these findings, future research and policy evaluation should model exposure as a dynamic pattern rather than a static, single-feature characteristic. Repeated assessment across adolescence could help identify whether different combinations and trajectories of exposure are differentially associated with problematic gaming, whether these associations vary across regulatory contexts, and which safeguards are most likely to reduce harm.

### 4.4 Offline leisure as a dynamic context rather than a static protective factor

With respect to RQ3, baseline participation in offline hobbies and perceived competence in extracurricular domains did not predict subsequent decreases in problematic gaming symptoms over one- and two-year follow-ups. These findings challenge a simple displacement assumption in prevention discourse—that greater engagement in offline leisure activities automatically protects against problematic gaming. Offline hobby participation was also socially patterned in this cohort, showing small positive associations with being enrolled in the academic track, living in a two-parent family, and reporting greater perceived financial comfort. This suggests that participation breadth may partly reflect differential access to structured activities rather than a uniformly available protective resource.

Importantly, these findings should not be interpreted as evidence that offline leisure contexts are irrelevant. Rather, they suggest that participation breadth alone may be too limited to capture whether offline leisure functions as a meaningful protective resource. Perceived competence in extracurricular domains likewise did not predict later symptom change, suggesting that this baseline, domain-general competence self-view did not operate as a detectable protective factor in the present models. Together, these results suggest that participation breadth and domain-general competence, as measured at baseline, may be too coarse to capture the qualities of offline activities that are most relevant to gaming-related risk.

One possible explanation is that offline activities are not protective simply because they occupy time that might otherwise be spent gaming, but because of what they provide in adolescents’ daily lives. Offline activities differ in their structure, social embeddedness, and the extent to which they are experienced as self-chosen rather than obligatory. If these qualities are limited—or if the activity does not match the social, emotional, or identity-related functions that gaming serves—simply “having hobbies” may not translate into fewer gaming-related problems over time. This interpretation is consistent with work suggesting that problematic online engagement may sometimes serve compensatory functions, for example by helping adolescents cope with psychosocial difficulties or negative mood states (Kardefelt-Winther, 2014). If so, substituting time is unlikely to matter unless offline activities provide adolescents with functions that are relevant to their everyday needs, including a felt sense of effectiveness, mastery, and psychological fulfilment, as emphasized in self-determination theory (Ryan and Deci, 2000).

From a harm-reduction perspective, these findings caution against prevention strategies that rely primarily on generic “get a hobby” messaging. A more promising approach may be to strengthen the developmental quality of adolescents’ everyday contexts by supporting socially embedded and meaningful activity settings, rather than assuming that increasing activity counts will by itself reduce risk (Coatsworth and Conroy, 2009; Denault and Poulin, 2009). In this view, the goal is not to replace video gaming with offline leisure, but to cultivate offline contexts that can meaningfully support adolescents’ psychological needs and overall wellbeing.

Future longitudinal work should therefore move beyond participation breadth to capture the level of engagement in each activity, including activity dose, continuity, setting quality, and the psychological functions that offline activities may serve for adolescents. Repeated measurement of offline contexts across adolescence could clarify how changes in activity involvement and experience relate to trajectories of problematic gaming over time.

### 4.5 Help-seeking as a preliminary recognition–action gap

Regarding RQ4, help-seeking for gaming-related difficulties was rare in this cohort, despite relatively frequent reports of knowing where to turn. This points to a descriptive gap between perceived help pathways and reported support use. Notably, only a small minority of those who reported help-seeking met the problematic gaming cut-off at T3, and none exceeded the cut-off at earlier waves. In addition, baseline problematic gaming did not prospectively predict later help-seeking: adolescents with higher symptom levels at T1 were not more likely to seek help at T3. Together, these findings suggest that reported knowledge of where help may be available and elevated symptom levels do not necessarily coincide with reported support use.

Help-seeking was captured at T3 through two broad indicators: whether adolescents reported knowing where they could turn for gaming-related difficulties, and whether they had ever sought such help. The knowledge item likely indexes a wide range of perceived help pathways, including parents, peers, school staff, or professional services, whereas the behavior item reflects a concrete step toward support without specifying the source, timing, or context of that support. This distinction helps contextualize why reporting knowledge of where to turn was substantially more common than reported help-seeking.

The finding that many adolescents reported knowing where to turn for help, while only a small minority reported actual help-seeking, challenges the assumption that low service use primarily reflects a lack of information. Broader youth mental health literature offers several plausible explanations for why perceived access may not translate into support use. Informational access is only one prerequisite for help-seeking, and factors such as low perceived need, stigma, uncertainty about problem severity, and privacy concerns may also inhibit action even when support options are known (Gulliver et al., 2010; Rickwood et al., 2007). In addition, adolescents may not always recognize or interpret gaming-related difficulties as warranting formal support (Jeong et al., 2018; Schneider et al., 2018). Gaming’s normative status in adolescent peer cultures may further reduce the perceived need for professional support by blurring when engagement becomes harmful (Griffiths, 2015). Even when difficulties are recognized, adolescents may also anticipate negative consequences of disclosure, or be uncertain about what services would actually offer, making silence or self-management appear safer than reaching out (Rickwood et al., 2007; Schneider et al., 2018).

From a harm-reduction perspective, the gap between perceived access to help and reported support use remains important because it suggests that informational awareness alone may be insufficient to promote early help-seeking. Given this gap, information provision alone is unlikely to be sufficient to increase uptake. More promising responses may therefore include low-threshold, non-stigmatizing forms of support in settings adolescents already inhabit, combined with brief screening and guidance that can help identify difficulties before they intensify (Karcher et al., 2023). Strengthening adult-mediated identification and referral may also be important, as help-seeking for adolescent behavioral and mental health concerns is often initiated or facilitated by parents, schools, and youth professionals rather than by adolescents themselves (Bore et al., 2025).

More promising ways to support earlier help-seeking may include embedding brief screening and guidance in settings adolescents already inhabit. In addition, services can normalize proportionate, non-pathologizing support options that do not require adolescents to self-identify as “in need of treatment.” Future research can sharpen the recognition–action gap by (1) distinguishing informal versus professional sources and self-initiated versus adult-mediated pathways, and (2) directly assessing perceived barriers and facilitators (e.g., stigma, anticipated restriction, confidentiality concerns) as well as perceived usefulness and fit of available support.

### 4.6 Implications for harm-reduction-oriented prevention

Taken together, these findings support a harm-reduction approach that is proportionate to observed risk and targets modifiable conditions rather than gaming time alone. Framed through an agent–set–setting lens, three priorities emerge.

*Agent*: Among the candidate predictors examined in this study, baseline engagement with gambling-like features provided the clearest prospective signal of increased problematic gaming symptoms at the one-year follow-up. At the same time, the broader pattern of associations in this study suggests that agent-level risk may be better understood not in terms of a single isolated mechanic, but in terms of adolescents’ exposure to combinations of gambling-like and monetized features over time. From this perspective, the more informative question is not which single mechanic is most harmful in isolation, but how the breadth, intensity, and combination of gambling-like affordances accumulate across adolescents’ gaming trajectories. Harm-reduction efforts may therefore benefit from focusing on broader modifiable features of monetized gaming environments, rather than on single mechanics in isolation.

*Setting:* Our findings do not support prevention approaches that treat offline leisure as an automatic substitute for gaming. Because baseline hobby participation was not prospectively associated with symptom decreases, prevention may benefit more from strengthening the quality and accessibility of adolescents’ everyday contexts than from promoting activity counts alone. The gap between knowing where to turn and actually seeking help likewise suggests that low-threshold support depends on accessible, adult-facilitated pathways. Parents, schools, and youth services may therefore play a key role in early identification, non-stigmatizing conversations, and referral options that do not require self-labeling as “in need of treatment.”

*Set:* Finally, the findings point to the importance of individual-level processes in whether gaming-related difficulties escalate or lead to support-seeking. In this cohort, baseline perceived competence in extracurricular domains was not associated with later symptom decreases, suggesting that perceived offline competence may not, by itself, capture the broader offline processes that could shape gaming-related risk. At the same time, the help-seeking findings indicate that symptom levels do not automatically translate into action, underscoring the importance of problem recognition, perceived need, and readiness to seek support.

### 4.7 Methodological considerations, limitations, and future directions

Several considerations frame the interpretation of these findings and point to clear next steps. First, key constructs were measured in ways that are pragmatic for school-based surveys but limit specificity. Gambling-like engagement was captured via an aggregated self-report index spanning co-occurring activities, which aligns with an ecosystem-oriented exposure concept but constrains inferences about individual mechanics and may be sensitive to recall and interpretation differences. Moreover, offline leisure and help-seeking were captured with pragmatic, brief indicators, which limits inference about activity quality, depth of engagement, and the nature and timing of support pathways. The extracurricular activity measure, for instance, indicated whether adolescents participated across activity domains, but did not capture frequency, duration, commitment, subjective involvement, or continuity of participation. Similarly, perceived competence was operationalized as a broad index across sports, music, and creative activities. Sensitivity analyses treating sport, music, and creative competence as separate predictors yielded substantively unchanged results, suggesting that the null finding for perceived offline competence was not driven by aggregation across domains. Accordingly, future longitudinal work should treat exposure, leisure, and help-seeking as dynamic, multi-dimensional constructs—capturing intensity, persistence, and change over time; qualitative features of settings; and barriers, pathways, and outcomes of support-seeking—ideally with repeated measurement and, where feasible, multi-informant perspectives (e.g., parents or school staff).

Second, the study was conducted in Flanders, a context with relatively high digital access and a distinctive regulatory environment regarding gambling-like game features. This supports ecological validity for similar settings but warrants caution when generalizing to regions with different market structures, cultural norms, policy regimes, extracurricular infrastructures, and school- or youth-based prevention systems. Longitudinal inference also relies on the subset retained over time, raising the possibility of selective attrition. If adolescents with higher symptom levels were less likely to participate in later waves, prevalence, persistence, and prospective associations may be underestimated. Cross-national longitudinal studies with harmonized measures and explicit modeling of attrition and policy context are therefore needed to examine how cumulative exposure interacts with regulatory and cultural environments to shape gaming-related risk.

Taken together, these considerations do not diminish the value of the findings; rather, they clarify what the present design can—and cannot—support and delineate a focused agenda for future research: stronger causal leverage, more context-sensitive exposure measurement, richer assessment of help-seeking pathways, and a more functionally grounded measurement of offline protective contexts.

## Data Availability

The datasets presented in this article are not readily available due to ethical and privacy restrictions related to sensitive data from adolescent participants. Requests to access the datasets should be directed to Maya Geudens at maya.geudens@kuleuven.be, subject to ethical approval and institutional permission.
